# Remapping Body Representation Using Virtual Reality in Chronic Neuropathic Pain: Systematic Review

**DOI:** 10.2196/71074

**Published:** 2025-06-03

**Authors:** Maria Grazia Maggio, Mirjam Bonanno, Andrea Calderone, Amelia Rizzo, Nebahat Bulut, Mahmood Bahramizadeh, Alessandra Benenati, Francesco Tomaiuolo, Angelo Quartarone, Daniela Floridia, Rocco Salvatore Calabrò

**Affiliations:** 1 IRCCS Centro Neurolesi Bonino Pulejo Messina Italy; 2 Department of Clinical and Experimental Medicine University of Messina Messina Italy; 3 Department of Anesthesiology Faculty of Medicine Istanbul Beykent University Istanbul Turkey; 4 University of Social Welfare and Rehabilitation Sciences Tehran Iran; 5 Department of Psychology University of Bologna Bologna Italy

**Keywords:** virtual reality, chronic neuropathic pain, patients with neurological conditions, remapping body representation, pain modulation, pain management

## Abstract

**Background:**

Chronic neuropathic pain (CNP) is a common consequence of neurological conditions such as spinal cord injury (SCI), complex regional pain syndrome (CRPS), and phantom limb pain (PLP). These conditions are often associated with distorted body representation (BR) and altered sensory processing. Virtual reality (VR) offers immersive, multisensory experiences that can modulate attention, recalibrate BR, and potentially alleviate pain.

**Objective:**

This systematic review aims to synthesize evidence on the use of VR-based interventions for managing CNP in patients with neurological conditions. It explores how VR can influence pain perception through body remapping. Furthermore, this review seeks to identify gaps in current research, offering recommendations for future research directions and clinical applications.

**Methods:**

We performed a comprehensive literature search in PubMed, Web of Science, and Scopus for studies published between January 2014 and December 2024. We included original studies that examined VR interventions in patients with neurological conditions and CNP, assessing pain reduction, improvements in BR, or functional recovery. We excluded reviews, animal model studies, migraine-related studies, and those lacking a clear VR intervention or relevant clinical outcome data. The quality of the included studies was evaluated using the revised Cochrane Risk of Bias Tool for Randomized Trials and the Cochrane Risk of Bias in Nonrandomized Studies of Interventions tool. Given the heterogeneity in study design, VR protocols, and outcome measures, a qualitative synthesis approach was adopted based on the synthesis without meta-analysis framework.

**Results:**

Ten studies—both randomized controlled trials and uncontrolled experimental designs—met the inclusion criteria. These studies focused on the application of VR in SCI (n=4, 40%), CRPS (n=4, 40%), and PLP (n=2, 20%), using interventions such as immersive VR, mirror visual feedback, visuotactile stimulation, and virtual body illusions. Sample sizes ranged from 9 to 70 participants, with varying degrees of neurological impairment. Most of the studies (n=7, 70%) reported substantial reductions in pain intensity and improvements in embodiment and perceived body ownership. In SCI, combining VR with neuromodulation techniques enhanced analgesic effects. In CRPS, modifying the visual appearance of the affected limb improved body image and decreased pain perception. In PLP, kinesthetic and visual feedback delivered through VR environments significantly reduced PLP and improved movement representation.

**Conclusions:**

Overall, the quality of evidence ranged from moderate to low, mainly due to small sample sizes, protocol heterogeneity, and risk of bias. Evidence regarding adherence to VR therapy was particularly limited. Nonetheless, VR shows strong potential as a noninvasive, patient-tailored therapeutic tool for CNP. VR could provide innovative and engaging strategies to reduce pain and enhance functional outcomes in populations with neurological conditions. Future research should focus on developing standardized protocols and conducting large-scale, high-quality trials to validate VR’s long-term efficacy and integration into routine clinical practice.

**Trial Registration:**

PROSPERO CRD42024606150; https://www.crd.york.ac.uk/PROSPERO/view/CRD42024606150

## Introduction

### Background

Chronic neuropathic pain (CNP) can derive from lesions and diseases involving the somatosensory nervous system [1]. This type of pain is usually chronic, that is, it either persists continuously or manifests with recurrent painful episodes, resulting from etiologically diverse disorders affecting the peripheral or the central nervous system [2,3]. In this context, CNP can be a common manifestation of various neurological diseases (eg, complex regional pain syndrome [CRPS], phantom limb pain [PLP], and spinal cord injury [SCI]), as patients may experience altered sensory processing and disruptions in body representation (BR) [4,5]. These pain experiences are frequently linked to the dysregulation of nociceptive pathways, including the spinothalamic, spinoreticular, and spinomesencephalic tracts, which contributes to increased pain sensitivity and persistent discomfort [4]. To better quantify these complex pain experiences, assessment tools such as the visual analog scale, McGill Pain Scale, and Numerical Rating Scale provide valuable information about the intensity and characteristics of pain (such as quality, location, and exacerbating and ameliorating factors), highlighting the complex and often severe impact on a patient’s quality of life [6]. A growing body of research supports the critical role of BR in pain perception for patients with neurological conditions [7-13]. BR refers to the cognitive and sensory mapping of the physical self, a process that is often distorted in neurological conditions [10]. This distortion can contribute to an intensified experience of pain, highlighting the need for therapeutic approaches that address this cognitive-sensory mismatch [14,15]. A clearer understanding of this connection emerges when considering that disruptions in the somatosensory and motor cortices can lead to a fragmented BR, where sensory inputs are misinterpreted, resulting in exaggerated or worsened pain symptoms [16-18]. This effect is associated with maladaptive neuroplasticity in cortical areas such as the primary somatosensory cortex, anterior cingulate cortex, and insular cortex, which together shape pain perception and intensity [10,14]. Building on this understanding, significant advancements in pain research have emerged from the introduction of the neuromatrix theory proposed by Melzack [19]. This model views pain as a complex experience shaped by sensory, emotional, and cognitive inputs within a biopsychosocial framework [19]. Within this framework, the neuromatrix, a broad neural network, processes pain by integrating physiological and psychological signals [19,20]. However, recent research has suggested that pain intensity can be dissociated from responses in the neuromatrix, emphasizing the importance of nonnociceptive factors such as emotions and anticipatory anxiety [21,22]. This perspective underscores that pain, particularly in its chronic neuropathic forms, is not merely a physiological reaction to tissue damage. On the contrary, it reflects a multifactorial experience shaped by the complex interaction of sensory, emotional, cognitive, and social dimensions. This multidimensional nature is evident in the phenomenon of distorted BR, which can amplify pain perception through maladaptive sensory processing and disrupted cognitive integration [10,13]. This disruption of BR often interacts with psychological constructs (ie, catastrophizing, anticipatory fear, and emotional distress), which can further intensify the pain experience, exacerbating symptoms beyond the initial nociceptive input [5,23]. In addition to these individual factors, cultural and social contexts influence how individuals interpret and express pain, further demonstrating its biopsychosocial complexity. This comprehensive approach is crucial for developing effective therapeutic strategies, as highlighted by the growing application of virtual reality (VR) to recalibrate BR and modulate pain perception through immersive and tailored interventions [19,24]. Indeed, psychological factors such as attention and cognitive interpretation are crucial in shaping pain perception; for instance, focusing on the painful stimulus tends to amplify pain perception, while distraction techniques can significantly reduce it [23,24]. In this context, interventions delivered through innovative technologies have proven effective in shifting attention away from pain, offering immersive environments that engage patients and diminish their perceived pain levels [25]. VR has emerged as a promising tool to fully immerse patients in simulated environments, using devices such as headsets and noise-canceling headphones to create interactive experiences [26]. This technology has proven effective for pain management, particularly in chronic pain scenarios, helping patients redirect their attention and alleviate perceived pain [27]. Indeed, VR offers a promising avenue to address these challenges in neurological rehabilitation [21,28]. By providing immersive and controlled environments tailored to individual sensory needs, VR interventions can stimulate motor and sensory processing to recalibrate BR [29]. This recalibration or “remapping” of the BR has shown the potential to modulate pain by altering nociceptive processing and sensory integration [24,30]. This effect provides the basis for understanding how VR can be leveraged in pain management. In fact, VR may have a positive impact on pain by modulating sensory experiences and perceptions, shifting attention, and recalibrating the internal body model [31]. Such “remapping” could play a crucial role in reducing pain intensity and improving functional outcomes in patients with neurological conditions. By facilitating the reorganization of BR, VR-based interventions help modulate pain perception through targeted alterations in sensory processing, offering new hope for managing CNP [31]. Moreover, research has shown that VR, combined with other psychological approaches such as relaxation and hypnosis, can optimize pain management, offering a noninvasive solution that integrates both physical and psychological aspects of the pain experience [24,26,27]. These distraction-based interventions have been especially effective for managing acute pain during medical procedures but also show potential for long-term treatment strategies [25]. This perspective underscores the need for an integrated approach to pain management.

### Objectives

Despite the promising potential of VR interventions in pain management, there is a lack of comprehensive evidence regarding their underlying mechanisms and long-term effectiveness in treating CNP in patients with neurological conditions. Specifically, the contribution of body remapping processes to pain modulation and functional recovery remains poorly investigated, particularly in relation to neurological conditions such as CRPS, PLP, and SCI. Thus, this systematic review aims to synthesize the existing evidence on the use of VR-based interventions for managing CNP in patients with neurological conditions. It focuses on exploring how VR can influence pain perception through mechanisms such as body remapping and improvements in BR, ultimately contributing to pain modulation and functional recovery. Furthermore, this review seeks to identify gaps in current research regarding the underlying mechanisms and long-term efficacy of VR interventions, while offering recommendations for future research directions and clinical applications.

## Methods

### Search Strategy and Eligibility Criteria

#### Overview

This systematic review aimed to investigate the effects of VR-based interventions on the management of CNP in patients with neurological conditions. The review protocol was registered on PROSPERO (CRD42024606150), ensuring methodological transparency and adherence to systematic review standards.

We conducted the review following the PRISMA (Preferred Reporting Items for Systematic Reviews and Meta-Analyses) 2020 guidelines [32]. The PRISMA checklist is presented in Multimedia Appendix 1.

We conducted a comprehensive literature search using the PubMed, Web of Science, and Scopus databases, covering studies published between January 2014 and December 2024. To ensure a systematic and reproducible search strategy, a combination of Medical Subject Headings (MeSH) terms and free-text keywords was used. Three distinct keyword combinations were applied across the databases:

- String 1: (all fields: “Nociceptive pathways”) AND (all fields: “Neuroplasticity”) AND (all fields: “Pain sensitivity”)
- String 2: (all fields: “Virtual reality”) AND (all fields: “Chronic pain”) AND (all fields: “Body awareness”)
- String 3: (all fields: “Body representation”) AND (all fields: “Pain perception”) AND (all fields: “Neurological patients”) AND (all fields: “Virtual reality”)

These distinct keyword combinations are summarized in Table 1.

**Table 1:** Database search strategies and keyword strings.

Databases	Medical Subject Headings (MeSH) terms and keyword strings
PubMed	(“Nociceptive pathways” AND “Neuroplasticity” AND “Pain sensitivity”) (“Virtual reality” AND “Chronic pain” AND “Body awareness”) (“Body representation” AND “Pain perception” AND “Neurological patients” AND “Virtual reality”)
Web of Science	(“Nociceptive pathways” AND “Neuroplasticity” AND “Pain sensitivity”) (“Virtual reality” AND “Chronic pain” AND “Body awareness”) (“Body representation” AND “Pain perception” AND “Neurological patients” AND “Virtual reality”)
Scopus	(“Nociceptive pathways” AND “Neuroplasticity” AND “Pain sensitivity”) (“Virtual reality” AND “Chronic pain” AND “Body awareness”) (“Body representation” AND “Pain perception” AND “Neurological patients” AND “Virtual reality”)

#### Population, Intervention, Comparison, and Outcomes Evaluation

We applied the Population, Intervention, Comparison, and Outcomes (PICO) model to define our search strategy. In particular, the population comprised patients with neurological diseases experiencing CNP, specifically those with altered BR and sensory processing issues. The intervention included the use of VR-based rehabilitation aimed at modulating sensory and motor processing to recalibrate BR and alleviate pain. The comparison involved standard pain management techniques or other non–VR-based rehabilitation methods (ie, nonpharmacological interventions and rehabilitation intervention without the use of VR). The outcomes included reduction in pain intensity, improved pain perception through body remapping, enhanced functionality, and improved quality of life for patients with neurological conditions and CNP.

#### Inclusion Criteria

To be included in this systematic review, studies needed to evaluate the potential of VR as a treatment for CNP. Studies had to involve interventions using VR to modify sensory experiences, such as body remapping and improved BR, for pain relief. They were required to provide quantitative or qualitative data on pain reduction, alterations in sensory experiences, or enhancements in BR. To maintain consistency in understanding, only studies published in English were considered. Eligible studies included original research such as randomized controlled trials (RCTs), nonrandomized experimental studies, cohort studies, and longitudinal studies that presented detailed information about the intervention, methodology, and outcome measures associated with VR use.

#### Exclusion Criteria

Studies that did not involve VR as part of the treatment for CNP and body perception were excluded. In addition, studies were excluded if they lacked comprehensive information about the VR intervention, such as its duration, technical parameters, or specific outcomes related to the targeted conditions. Only articles published in English were included because reliable translation for non-English studies could not be ensured, which could lead to misinterpretation. Reviews, including literature reviews, systematic reviews, integrative reviews, and narrative reviews, were excluded from direct analysis, although their reference lists were screened to identify relevant primary studies. Research conducted on animal models or populations not experiencing CNP was excluded to maintain the review’s focus on human populations with relevant conditions. Studies focusing on migraines were also excluded. While migraines are a form of chronic pain, their neurovascular pathophysiology and therapeutic approaches differ significantly from the mechanisms underlying VR interventions aimed at “remapping” BR, which is central to this review.

### Selection Process

Two reviewers (MGM and AC) conducted searches independently using Boolean operators and controlled vocabulary such as MeSH terms to ensure comprehensive and accurate identification of relevant studies.

The risk of bias in RCTs was assessed using the revised Cochrane Risk of Bias Tool for Randomized Trials (RoB 2) [33], while the Cochrane Risk of Bias in Nonrandomized Studies of Interventions (ROBINS-I) tool [34] was used for the nonrandomized studies included in this review. In addition, the overall quality of evidence for each outcome was evaluated using the GRADE (Grading of Recommendations, Assessment, Development, and Evaluation) framework [35], which considers factors such as risk of bias, inconsistency, indirectness, imprecision, and publication bias to provide a comprehensive evaluation of the strength of evidence.

The 2 reviewers (MGM and AC) independently screened titles, abstracts, and full texts of all articles. Any discrepancies during this process were resolved by consulting a third reviewer (M Bonanno), who made the final decision. Extracted data included information on study design, the number of participants, participant characteristics, neurological disorders and pain types, details of the VR rehabilitation intervention (eg, duration and type), outcomes evaluated, and key findings. To minimize bias (eg, missing results bias, publication bias, time lag bias, and language bias), the reviewers conducted cross-validation of the extracted data.

Agreement between the 2 reviewers (MGM and AC) was assessed using the κ statistic, with a score indicating substantial agreement (>0.61) demonstrating the reliability of the screening and data extraction process. All articles meeting the inclusion criteria were thoroughly reviewed and summarized, with key topics identified based on predefined inclusion and exclusion criteria.

### Data Collection Process

To synthesize the available data, a qualitative analytical approach was applied due to the substantial heterogeneity among the included studies. This heterogeneity was evident in various aspects, including the study designs, such as RCTs and observational studies; the VR intervention protocols, including immersive VR, visual feedback, and biofeedback; and the outcome measures used, such as pain reduction, changes in BR, functional improvements, and quality of life.

Given this methodological diversity, it was not feasible to conduct a meta-analysis, which requires a sufficient level of similarity across studies in terms of design, intervention type, and measured outcomes to ensure meaningful statistical comparison. To address this limitation and ensure methodological rigor, the synthesis was structured according to the synthesis without meta-analysis framework [36]. This approach promotes transparency and consistency in data interpretation, particularly when quantitative synthesis is not possible.

The qualitative synthesis aimed to identify key themes, recurring patterns, and discrepancies across the studies, focusing on outcomes related to pain reduction, the recalibration of BR, and improvements in functional outcomes and quality of life. Studies were categorized based on the type of VR intervention, patient characteristics, and the specific outcome domains addressed in the research.

The results were systematically presented in tabular form, summarizing key study characteristics, intervention details, and measured outcomes. This structured synthesis provided a comprehensive understanding of the potential effectiveness of VR-based interventions in managing CNP in patients with neurological conditions. In addition, the findings were critically evaluated to highlight existing gaps in the literature, address methodological limitations, and suggest specific directions for future research.

## Results

### Overview

**Figure 1:**
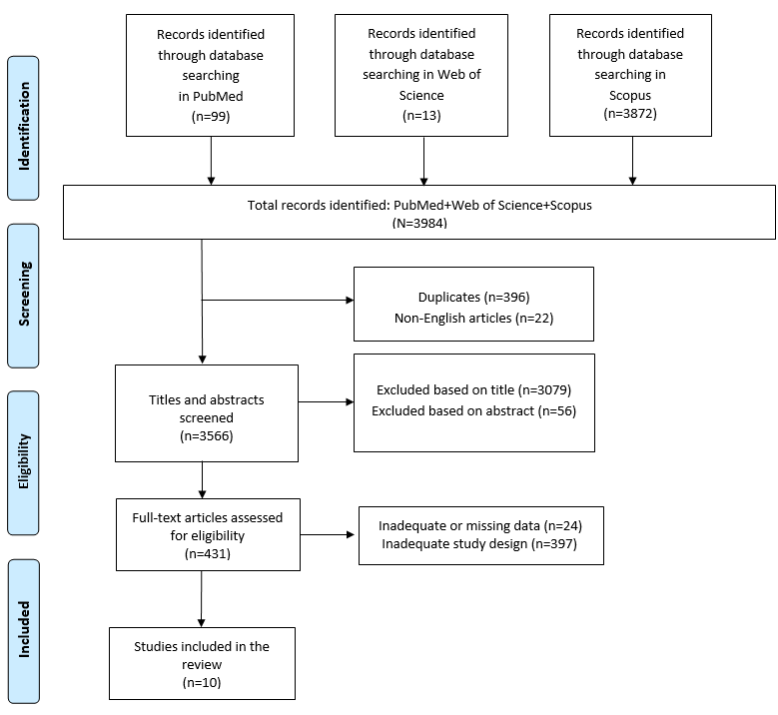
PRISMA (Preferred Reporting Items for Systematic Reviews and Meta-Analyses) 2020 flow diagram of evaluated studies.

We identified 3984 articles, of which 396 (9.94%) were excluded after screening because they were duplicates, 22 (0.55%) were excluded because they were not published in English, and 3135 (78.69%) were excluded based on title and abstract screening. Finally, of the remaining 431 articles, 421 (97.7%) were removed based on screening for inadequate and untraceable study designs (refer to the PRISMA flow diagram [37] in Figure 1), and 10 (2.3%) articles that met the inclusion criteria were included in the review. These studies are summarized in Tables 2 and 3.

**Table 2:** Characteristics of studies included in the review.

Study	Aim	Study design	Sample size and characteristics	Outcome measures
Pozeg et al [38], 2017	To examine how the sensations and pain levels of individuals with SCI^a^ are impacted by experiencing virtual leg illusion and full-body illusion	RCT^b^	40 individuals (20 with SCI and 20 healthy controls) Age and sex: the 20 participants with SCI included 2 female individuals and ranged in age from 23 to 71 (mean 47.3, SD 12.0) y; the 20 healthy participants included 2 female individuals and ranged in age from 23 to 70 (mean 43.0, SD 11.8) y	Questionnaire for illusion intensity, VAS^c^, and CDS^d^
Lewis et al [8], 2021	To assess whether using the MIRAGE system (a noninvasive MVR^e^ system) for a visual illusion intervention enhances body perception, ownership, and liking of the affected hand and decreases pain in patients with CRPS^f^	RCT	45 participants assigned randomly: 23 in the manipulation group and 22 in the control group Age: mean 52 (SD 13) y Sex (female): 29 (64%)	BPDS^g^, NRS^h^, NPSI^i^, and perceptual statement ratings (eg, ownership and satisfaction with hand appearance)
Hwang et al [7], 2014	To assess how BPD^j^ and pain intensity in patients with CRPS are impacted by 3 treatments: VBS^k^, WM^l^ only, and MR^m^	RCT	39 patients Age: mean 36.31 y (VBS); mean 43.00 y (WM); mean 43.08 y (MR) Sex: predominantly male (92.3% in VBS group; 61.5% in WM and MR groups)	BPD assessed using the updated Body Perception Disturbance Questionnaire and pain severity ranked on a Likert scale
Tabacof et al [39], 2024	To assess the practicality and efficiency of virtual settings (scenic, somatic, and control) for managing CNP^n^ in people with SCI	RCT	32 participants Age: adults aged ≥18 y Sex (male): 15 (68%)	NPSI, NPS^o^, SCIM III^p^, BDI^q^, ITQ^r^, PQ^s^, UES^t^, and PGIC^u^
Scandola et al [11], 2020	To investigate the effects of passive movement and VR^v^ visual cues on body perception and physiological responses in individuals with SCI compared to those who are healthy	Uncontrolled experimental study	70 individuals (19 with complete paraplegia, 23 with incomplete paraplegia, and 28 healthy controls) Age: 40-45 y (mean) Sex: 13 female individuals and 57 male individuals	Physiological responses (skin conductance, pulse pressure waves, and respiration), BPQ^w^ and CCE^x^
Ichinose et al [40], 2017	To assess how well a neurorehabilitation system using VR and sensory feedback works in decreasing PLP^y^ in individuals with brachial plexus avulsion or arm amputation, while improving body perception	Uncontrolled experimental study	9 patients Age: 43-75 y Sex: 1 female individual and 8 male individuals	NRS for pain, SF-MPQ^z^, and evaluation of referred sensations
Osumi et al [41], 2019	To examine how VR-MVF^aa^ rehabilitation impacts the intensity of PLP and the movements of phantom limbs in individuals with amputations and patients with brachial plexus avulsion	Uncontrolled experimental study	19 patients Age: mean 48.10 (SD 11.31) y Sex: 5 female individuals and 14 male individuals	NPSI, SF-MPQ, and BCT^ab^
Matamala-Gomez et al [9], 2019	To examine the impact of different morphological traits (transparency and size) of a virtual arm on the way patients with upper limb CNP perceive pain, particularly in comparing patients with CRPS type I and those with peripheral nerve injury	Uncontrolled experimental study	19 patients Age: 40-55 y Sex: 14 female individuals and 5 male individuals	NRS, MMSE^ac^, and FAB^ad^
Solcà et al [12], 2021	To explore whether adding a VR system that shows the tingling sensation caused by SCS^ae^ can improve pain relief	Uncontrolled experimental study	15 patients Age: mean 47.7 (range 33-61) y Sex: 5 female individuals and 10 male individuals	VAS
Solcà et al [42], 2018	To assess how HEVR^af^ impacts pain, embodiment, and upper limb function in patients experiencing CRPS after trauma or stroke to the upper limb	Uncontrolled experimental study	24 patients with CRPS and 24 age- and sex-matched healthy controls Age: 27-80 (mean 50.3) y Sex: 14 female individuals and 10 male individuals in the patient group; 14 female individuals and 10 male individuals in the control group	Brief Pain Inventory, VAS, 9-Hole Peg Test, Jamar grip strength test, proprioceptive drift, 10-item questionnaire on virtual hand ownership, and heart rate (measured via frequency-domain analysis)

^a^SCI: spinal cord injury.

^b^RCT: randomized controlled trial.

^c^VAS: visual analog scale.

^d^CDS: Cambridge Depersonalization Scale.

^e^MVR: mediated virtual reality.

^f^CRPS: complex regional pain syndrome.

^g^BPDS: Body Perception Disturbance Scale.

^h^NRS: Numerical Rating Scale.

^i^NPSI: Neuropathic Pain Symptom Inventory.

^j^BPD: body perception disturbance.

^k^VBS: virtual body swapping.

^l^WM: watching movement.

^m^MR: mental rehearsal.

^n^CNP: chronic neuropathic pain.

^o^NPS: Neuropathic Pain Scale.

^p^SCIM III: Spinal Cord Independence Measure III.

^q^BDI: Beck Depression Inventory.

^r^ITQ: Immersive Tendencies Questionnaire.

^s^PQ: Presence Questionnaire.

^t^UES: user experience score.

^u^PGIC: Patient Global Impression of Change.

^v^VR: virtual reality.

^w^BPQ: Body Perception Questionnaire.

^x^CCE: crossmodal congruency effects.

^y^PLP: phantom limb pain.

^z^SF-MPQ: Short-Form McGill Pain Questionnaire.

^aa^VR-MVF: virtual reality–based mirror visual feedback.

^ab^BCT: bimanual circle-line coordination task.

^ac^MMSE: Mini-Mental State Examination.

^ad^FAB: frontal assessment battery.

^ae^SCS: spinal cord stimulation.

^af^HEVR: heartbeat-enhanced virtual reality.

**Table 3:** Results of studies included in the research.

Study	Intervention duration	Main findings	Effect size and certainty of evidence	VR^a^ intervention and body remapping
Pozeg et al [38], 2017	Each session lasted 60 s per condition	Synchronous visual and tactile stimulation greatly boosted perceived ownership and feelings of touch Patients with SCI^b^ had less of a sense of ownership over their legs compared to the control group but reported similar sensations of touch Pain decrease was noted exclusively during the synchronous condition for lower-back stimulation, although this result was not maintained after adjusting for multiple comparisons	Effect size: significant results were found with P values indicating significance for different variables (eg, synchrony and ownership: P=.04; synchrony and touch: P=.008) Certainty of evidence: moderate, backed by strong statistical analyses yet hindered by the small sample size and the absence of correction for certain comparisons	The VR protocols used visuotactile synchrony to create ownership illusions for virtual legs and a virtual body; the study demonstrated that cortical reorganization can be influenced after SCI, indicating that synchronized stimulation close to the injury site may affect sensory perception and possibly help reduce neuropathic pain Device: head-mounted display Setting: clinical environment, with participants seated in wheelchairs
Lewis et al [8], 2021	Attendees took part in 4 intervention meetings across 4 wk and 1 follow-up meeting held 2 wk after the last intervention	The control group experienced a higher level of BPD^c^ and pain intensity than the manipulation group after both single and repeated interventions The therapeutic effects were sustained over time through repeated exposure Customized changes in appearance played a vital role in ensuring participant contentment and therapeutic advantages	Effect size: effect sizes were determined by dividing the average group difference by the combined SD, using a 95% CI Certainty of evidence: the study followed strict randomized controlled trial guidelines, such as randomization, blinding of participants, and obtaining ethics approvals; yet, the reduced sample size could impact confidence in the findings	The MIRAGE system, a noninvasive MVR^d^ device, was used in the intervention to digitally change the look of the impacted hand, resulting in enhancements in body perception and pain relief Device: MIRAGE system Setting: laboratory-controlled setting
Hwang et al [7], 2014	Single session	The VBS^e^ group exhibited marked progress in BPD, particularly in ownership, temperature, pressure, and the inclination to cut off the limb There were no notable differences noted in the BPD or pain intensity levels between the WM^f^ and MR^g^ groups	Effect size: moderate effect size (Cohen d=0.16) found for the group×time interaction in BPD Certainty of evidence: moderate because of the limited sample size and the absence of notable impacts in certain groups	The treatment consisted of giving patients corrective visual feedback through matching their movements with a virtual body shown on a VR device; this blend of visual and mental movements involving sensory, motor, and cognitive input was designed to restore the affected limb and enhance body awareness, ultimately decreasing BPD Device: VR2000 3D visor head-mounted display Setting: tertiary pain management center located in Seoul, South Korea
Tabacof et al [39], 2024	8 wk, including 4 wk of intervention (12 sessions) and a 4-wk follow-up	Virtual settings, particularly those centered on somatics in VR, notably diminished CNP^h^ (overall NPSI^i^ scores) in contrast to the control group Enhancements in pain-related quality of life (PGIC^j^) and user satisfaction (UES^k^) were observed Participants indicated a favorable experience with VR environments, endorsing its practicality for clinical or telehealth applications	Effect size: not specified Certainty of evidence: moderate, based on the pilot nature of the trial and robust randomization and blinding procedures	The VR intervention in this study encompassed both scenic and somatic virtual settings, along with a control condition; the scenic settings showcased engaging 360-degree vistas, including woodlands, shorelines, and galleries, with vibrant alterations, depending on the user’s head movements Device: in the clinic sessions, HTC Vive headsets were used, providing a premium immersive experience within a controlled setting; home sessions used Destek V5 headsets paired with smartphones, enabling participants to connect on the web through a selected YouTube VR playlist Setting: in the clinic and at home
Scandola et al [11], 2020	Participants underwent 2 experimental sessions separated by a washout period ranging from 81 to 257 d	The groups of individuals with SCI exhibited varying reactions to tactile and visual stimuli in comparison to the control group The passive motion videos had varying effects on physiological and perceptual responses among different groups The BPQ^l^ showed differences in how groups experienced body awareness subjectively	Effect size: physiological and perceptual differences between the groups showed a moderate effect size (Cohen f=0.25) Certainty of evidence: moderate	The VR intervention aimed to investigate how participants perceived movement in their legs while watching videos of either passive leg movements (vision: mobilization) or immobile legs (vision: no mobilization); the videos were shown from a first-person viewpoint, and each lasted 2 min; the study concentrated on how visual and tactile cues impacted the participants’ sense of body awareness and perception Device: Oculus Rift DK1 head-mounted display Setting: laboratory environment where participants were seated comfortably in a chair, and their feet were placed in designated foot compartments attached to a wooden frame
Ichinose et al [40], 2017	2-4 d	The study discovered that tactile feedback, especially from the cheek condition, greatly decreased pain in patients with PLP^m^ Some participants reported referred sensations, indicating better body perception, which helped improve the rehabilitation process	Effect size: the pain reduction effect size ranged from moderate to large (r=0.68 for cheek condition vs no stimulus condition; r=0.68 for cheek condition vs intact hand condition); the size of the effect in reducing sensory pain was greater in the cheek condition Certainty of evidence: moderate, as there were significant results with some variation in the pain reduction responses across participants and conditions	The VR system used visual and tactile feedback to improve alignment between perceived and real limb movements, a technique proven to help with pain perception and assist in body image reconstruction Device: Oculus Rift immersive head-mounted display and Kinect sensor to track the movements of the intact limb Setting: quiet room within the Anesthesiology and Pain Relief Center at the University of Tokyo Hospital
Osumi et al [41], 2019	Single-session, 20-min VR-MVF^n^ rehabilitation	VR-MVF rehabilitation led to a notable enhancement in phantom limb mobility and reduction in PLP The virtual phantom limb’s feeling of reality was linked to improvements in phantom limb movement and relief from PLP	Effect size: large effects were indicated by effect sizes of 0.87 for phantom limb movement improvement and 0.83 for PLP alleviation (SF-MPQ^o^) Certainty of evidence: high level of confidence, backed by statistical significance (P<.001) and substantial effect magnitudes	The VR intervention intended to reconfigure the perception of the missing limb by displaying a mirrored virtual representation of the unaffected limb, enabling individuals to observe and manage the movements of their virtual phantom limb using the motion of their actual limb Device: Oculus Rift for immersive feedback; the system also used Kinect for Windows v2 and Leap Motion to detect and capture the movements of the intact arm and hand Setting: clinical outpatient setting at the University of Tokyo Hospital
Matamala-Gomez et al [9], 2019	Single 55-min session	The pain perception of patients with CRPS^p^ type I and those with peripheral nerve injury was influenced by the transparency and size of the virtual arm, as different levels of transparency and arm size impacted the pain ratings These virtual changes also influenced the sensory perception of the arm, specifically in terms of body ownership	Effect size: not specified Certainty of evidence: moderate	The virtual body was used to create a feeling of possessing the virtual arm, along with combining visual and tactile stimuli to strengthen the sense of owning the body Device: Oculus Rift Development Kit 2, a head-mounted display with a resolution of 960×1080 pixels per eye and a field of view of 100°, presented at 75 Hz Setting: pain unit at Hospital Clínic de Barcelona, where patients participated in a single 55-min experimental session
Solcà et al [12], 2021	2 experimental sessions conducted 24 h apart	A substantial decrease in pain when using SCS^q^-VR resulted in lower pain levels after the stimulation period A slight decrease in pain was noticed in the incongruent SCS-VR condition, whereas no pain relief was seen in the VR-only condition	Effect size: significant pain reduction (t14=−4.11; P=.001) for congruent SCS-VR; moderate reduction for incongruent SCS-VR (t14=−1.99; P=.07) Certainty of evidence: moderate	The visual feedback helped patients “remap” their body sensations by aligning the visual experience with the physical sensations (paresthesia) that they felt from the SCS, which was hypothesized to enhance the analgesic effects Device: Oculus Rift with a 2160×1200 display resolution, 110-degree field of view, and 90-Hz refresh rate; the device included motion tracking via accelerometer, gyroscope, and magnetometer Setting: physiotherapy room where patients experienced a VR scenario customized to their sensory feedback from the SCS
Solcà et al [42], 2018	Every individual experienced 3 sets of synchronized and unsynchronized situations during a single session lasting approximately 90 min	Synchronous HEVR^r^ decreased pain perception and enhanced embodiment in patients with CRPS, whereas asynchronous conditions did not have a notable impact	Effect size: Hedges g=0.99 (indicating a large effect size for pain reduction and embodiment improvements) Certainty of evidence: high due to the controlled design, suitable statistical analysis, and strong effect size	The study used VR treatments to evaluate body remapping in individuals with CRPS after upper limb injury or stroke Device: Oculus DK1 Setting: clinical setting, specifically at the Department of Orthopedic Surgery and the Department of Clinical Neuroscience at Geneva University Hospital, as well as the Hand Rehabilitation Unit at Sion Clinique Romande de Réadaptation

^a^VR: virtual reality.

^b^SCI: spinal cord injury.

^c^BPD: body perception disturbance.

^d^MVR: mediated virtual reality.

^e^VBS: virtual body swapping.

^f^WM: watching movement.

^g^MR: mental rehearsal.

^h^CNP: chronic neuropathic pain.

^i^NPSI: Neuropathic Pain Symptom Inventory.

^j^PGIC: Patient Global Impression of Change.

^k^UES: user experience score.

^l^BPQ: Body Perception Questionnaire.

^m^PLP: phantom limb pain.

^n^VR-MVF: virtual reality–based mirror visual feedback.

^o^SF-MPQ: Short-Form McGill Pain Questionnaire.

^p^CRPS: complex regional pain syndrome.

^q^SCS: spinal cord stimulation.

^r^HEVR: heartbeat-enhanced virtual reality.

### Quality of Included Studies: Risk of Bias

We assessed the risk of bias using appropriate tools based on the design of the included studies [7-9,11,12,38-42].

#### RoB 2 Tool Evaluations

**Figure 2:**
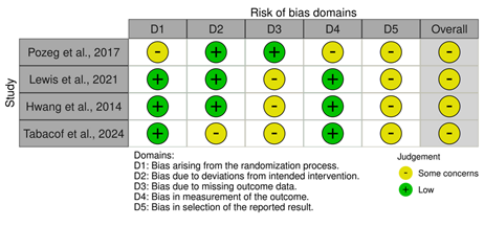
Risk-of-bias assessment of the randomized controlled trial studies using the revised Cochrane risk-of-bias tool for randomized trials.

Of the 10 studies, 4 (40%) RCTs [7,8,38,39] were evaluated with the RoB 2 tool [33] (Figure 2) across 5 domains:

- D1: bias arising from the randomization process
- D2: bias due to deviations from intended interventions
- D3: bias due to missing outcome data
- D4: bias in measurement of the outcome
- D5: bias in selection of the reported result

The RoB 2 analysis highlighted the study by Lewis et al [8] as the most methodologically robust study, demonstrating a low risk of bias across all domains except for D4 (bias in measurement of the outcome), where some concerns were identified. By contrast, Pozeg et al [38] and Hwang et al [7] showed recurring issues in D1 (bias arising from the randomization process), D4, and D5 (bias in selection of the reported result), suggesting potential weaknesses in randomization processes, measurement practices, and reporting transparency. All studies demonstrated a low risk of bias in D2 (bias due to deviations from intended interventions) and D3 (bias due to missing outcome data), reflecting adherence to intervention protocols and appropriate handling of missing data. The common issues observed in D4 highlight the need for more consistent and unbiased measurement strategies, while concerns in D1 and D5 underline the importance of robust randomization and prespecification of outcomes. However, the study by Tabacof et al [39] presented low risk in D1 (randomization process) and D4 (outcome measures) but reported some concerns in D2, D3, and D5, showing reduced control for bias in intervention-related domains, potential bias due to missing outcome data, and the presentation of the results. Overall, the study by Lewis et al [8] emerged as the strongest, while the findings from the studies by Pozeg et al [38], Hwang et al [7], and Tabacof et al [39] should be interpreted cautiously due to the identified methodological limitations.

#### ROBINS-I Tool Evaluations

**Figure 3:**
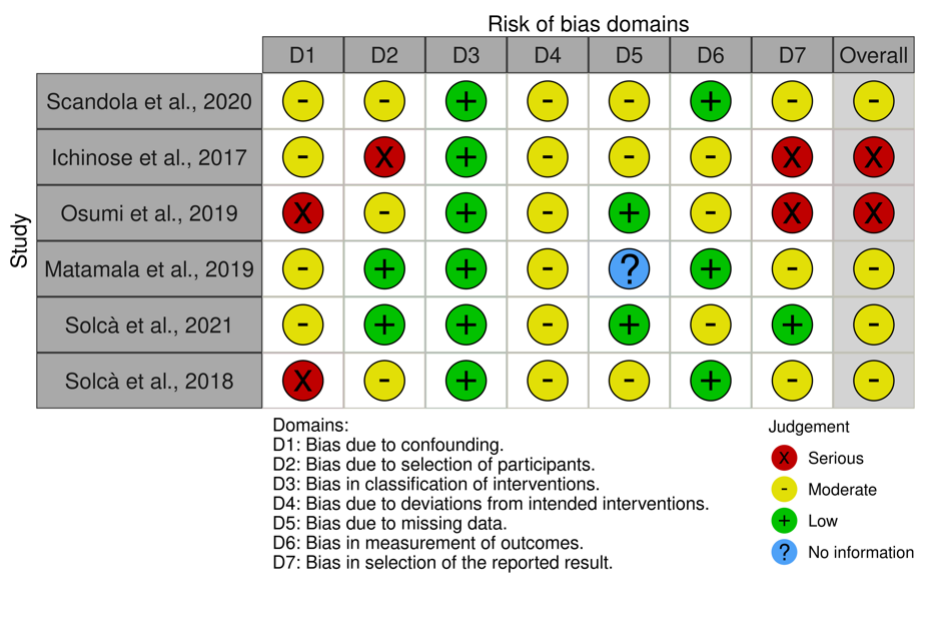
Risk-of-bias assessment of the nonrandomized experimental studies using the Cochrane Risk of Bias in Nonrandomized Studies of Interventions tool.

For the nonrandomized experimental studies (6/10, 60%) [9,11,12,40-42], we applied the ROBINS-I tool (Figure 3) [34], which assesses bias in 7 domains:

- D1: bias due to confounding
- D2: bias in participant selection
- D3: bias in classification of interventions
- D4: bias due to deviations from intended interventions
- D5: bias due to missing data
- D6: bias in outcome measurement
- D7: bias in selection of the reported outcome

The analysis showed a diverse range of methodological quality in various fields. The studies by Matamala-Gomez et al [9] and Solcà et al [12] exhibited the highest methodological rigor, with predominantly low-risk judgments across domains and no serious concerns. By contrast, the studies by Osumi et al [41] and Solcà et al [42] showed substantial weaknesses, with serious risks in D1 (bias due to confounding) and D7 (bias in selection of the reported outcome), affecting their overall reliability. The study by Ichinose et al [40] displayed serious concerns in D2 (bias in participant selection) and D7, indicating challenges with participant recruitment and outcome reporting. Common methodological issues were observed in D1 (confounding; 6/6, 100%) and D7 (selection of the reported outcome) across most of the studies (5/6, 83%), except for the study by Matamala-Gomez et al [9], which managed to avoid these risks. Moderate concerns in D4 (bias due to deviations from intended interventions) were also noted in several of the studies (6/6, 100%), reflecting potential inconsistencies in intervention protocols. The study by Scandola et al [11] showed mixed results, with moderate risks in D1, D2, and D5 (bias due to missing data), indicating limitations in controlling confounders, participant selection, and data handling. Overall, while studies such as the ones by Matamala-Gomez et al [9] and Solcà et al [12] demonstrated strong methodological quality, others, such as the ones by Osumi et al [41] and Solcà et al [42], revealed substantial biases, particularly in confounding and outcome reporting. Overall, the quality varied substantially, with the studies by Matamala-Gomez et al [9] and Solcà et al [12] standing out as methodologically sound and providing reliable evidence, while other studies faced notable challenges that limited the robustness of their findings.

#### Overall Quality of Studies

In addition to assessing the risk of bias using the RoB 2 tool for RCTs and the ROBINS-I tool for nonrandomized studies, we evaluated the overall quality of evidence using the GRADE framework. The GRADE approach considers 5 key factors: risk of bias, inconsistency, indirectness, imprecision, and publication bias.

For each outcome, we assigned a quality rating (high, moderate, or low) based on these criteria. The final assessment is summarized in Table 4.

The GRADE assessment indicated variability in the overall quality of evidence across different outcomes. The quality of evidence for pain reduction was generally moderate, reflecting a mix of well-conducted RCTs and nonrandomized studies, with some concerns regarding risk of bias and imprecision. Evidence for improvements in BR and functional outcomes was more heterogeneous, with lower overall ratings due to study design limitations and variability in intervention protocols. The weakest evidence was associated with adherence to VR therapy, where most of the studies (6/10, 60%) were observational and exhibited high risk of bias and inconsistency.

The evaluation of evidence strength was based on standardized GRADE criteria, considering study design, sample size, risk of bias, and the reproducibility of results (Table 4).

**Table 4:** GRADE (Grading of Recommendations, Assessment, Development, and Evaluation) evaluation.

Outcome	Study design	Risk of bias	Inconsistency	Indirectness	Imprecision	Publication bias	Overall quality of evidence
Pain reduction	RCTs^a^ and non-RCTs	Moderate	Moderate	Low	Moderate	High	Moderate
Improved body representation	RCTs and observational studies	High	High	Moderate	High	High	Low
Functional improvement	RCTs and cohort studies	Moderate	Moderate	Low	Moderate	Moderate	Moderate
Adherence to VR^b^ therapy	Observational studies and case reports	High	High	High	High	High	Low

^a^RCT: randomized controlled trial.

^b^VR: virtual reality.

### Synthesis of Evidence

#### Overview

We synthesized studies on the application of VR-based interventions combined with sensory feedback to address key challenges in the management of CNP in neurological disorders. These interventions focused on recalibrating BR, modulating pain experience, and improving embodiment. The 10 selected studies included 4 (40%) on managing CNP and SCI [11,12,38,39], 4 (40%) on modulating BR in CRPS and chronic pain [7-9,42], and 2 (20%) on VR-based BR for PLP and amputation-related pain [40,41]. These studies were diverse in design, populations, and methodologies, providing a comprehensive perspective on the application of VR for chronic pain management. The sample sizes ranged from 9 to 70 participants, with different age groups and both sexes represented, although male participants predominated in some of the CRPS studies (4/10, 40%) [7]. Interventions primarily involved RCTs or uncontrolled experimental studies, with intervention durations ranging from 60 seconds to 90 minutes and intervention periods ranging from a single session to several weeks. Outcomes were assessed using instruments for pain intensity (eg, visual analog scale and Numerical Rating Scale), BR, embodiment, and ownership. The results showed substantial pain reduction and improved embodiment, particularly with interventions that used synchronous sensory feedback. In individuals with amputations, VR substantially reduced PLP, while patients with CRPS experienced improved BR and ownership of the virtual limbs.

While the results demonstrated medium to high certainty, limitations (ie, small sample sizes, short intervention durations, and insufficient adjustment for multiple comparisons) potentially impacted generalizability. However, by addressing the cognitive-sensory mismatch underlying pain, the studies highlighted that VR may represent a promising therapeutic innovation that integrates neuroscience and technology to improve patient outcomes.

#### VR-Based Rehabilitation Interventions for CNP and SCI

Of the 10 studies, 4 (40%) highlighted the potential benefits of using VR and multisensory integration to enhance pain management and BR among individuals with SCI and CNP [11,12,38,39]. These studies implemented innovative neurorehabilitation protocols that integrated VR with neuromodulatory techniques or passive motion to influence body ownership, peripersonal space (PPS), and chronic pain.

In an RCT, Pozeg et al [38] investigated body ownership and pain regulation through a virtual leg illusion and full-body illusion in 20 patients with SCI and paraplegia and 20 healthy controls. The patients with SCI exhibited reduced reactions to multisensory stimuli that created a sense of leg ownership compared to controls, with leg ownership declining further as the time since injury increased. Notably, global body ownership did not differ significantly (*P*=.03) between the groups. Mild pain relief was observed only when visuotactile stimulation was applied simultaneously to the lower back during the virtual leg illusion. These findings suggest that VR-based interventions targeting specific body parts could enhance body ownership and alleviate pain in patients with SCI by leveraging multisensory inputs [38].

An uncontrolled experimental study examined how PPS was represented in 30 patients with paraplegia and SCI (with both complete and incomplete lesions) as well as in healthy controls. The results indicated that residual motor feedback, provided through immersive VR videos during passive movement, was crucial for restoring PPS near the feet in patients with paraplegia. By contrast, healthy individuals showed PPS suppression when faced with visual and motor mismatches. Patients with incomplete lesions demonstrated greater PPS restoration when they had higher interoceptive sensitivity, underscoring the role of residual motor responses and internal BR in PPS rehabilitation for patients with SCI [11]. Moreover, Solcà et al [12] tested a novel approach combining spinal cord stimulation (SCS) with personalized VR feedback in 15 individuals with chronic leg pain and SCS implants. When SCS-induced paresthesia was paired with congruent VR feedback, pain ratings dropped by 44%, outperforming incongruent SCS-VR or VR alone. Repeated sessions enhanced these analgesic effects, which persisted beyond the stimulation period, indicating a carryover benefit. This method highlights the synergistic potential of VR and neuromodulation in delivering targeted, durable pain relief for chronic pain management [12]. Finally, Tabacof et al [39] examined the viability of virtual environments for addressing CNP among patients with SCI. The researchers randomly assigned 32 participants to scenic, somatic, and control VR groups, achieving a completion rate of 68.75%. The main results (Neuropathic Pain Symptom Inventory scores) indicated notable enhancement in both somatic and scenic VR groups compared to the control group. The secondary outcomes, such as depression and functionality, emphasized improved well-being and autonomy. The research confirmed the potential of VR as a noninvasive, scalable method for managing CNP [39].

#### VR Applications and BR Modulation in CRPS and Chronic Pain Conditions

The reviewed studies highlighted the potential of VR-based rehabilitation interventions for managing pain in individuals with CRPS and chronic arm pain, particularly those experiencing disruptions in BR and sensory processing. Despite variations in VR methodologies across these studies, all demonstrated the significant impact of VR on the sensory and perceptual aspects of pain, reinforcing its potential as a nonpharmacological therapeutic approach.

Indeed, Lewis et al [8] conducted an RCT that investigated 45 patients with treatment-resistant CRPS and body perception disturbance (BPD) in their upper limbs. In the experimental group, patients viewed a digital image of their affected hand through VR, modified to appear as they desired, while the control group saw unchanged images. The findings revealed a notable decrease in BPD after just 1 session. Furthermore, the subgroup receiving multiple interventions consistently experienced significant pain reduction. These results suggest that changing the visual appearance of the painful limb can successfully reduce pain and enhance BR [8]. Similarly, Hwang et al [7] explored immersive techniques by introducing virtual body swapping with mental rehearsal to 39 patients with CRPS. While overall pain levels remained stable across the groups, only those in the virtual body swapping condition showed improvements in BPD, suggesting that immersive, realistic VR experiences can uniquely target BR disturbances.

A different approach was adopted by Solcà et al [42], who combined VR with neuromodulation in a double-blind crossover study of 24 patients with CRPS. By synchronizing VR feedback with the patient’s heartbeat (heartbeat-enhanced VR), they achieved significant (*P*=.001) reductions in pain and improvements in motor skills [42]. The absence of these effects in asynchronous conditions and healthy controls underscores the specificity of heartbeat-enhanced VR, highlighting the potential for integrating physiological signals into VR to deliver personalized, targeted therapies.

Finally, Matamala-Gomez et al [9] examined how modifying the transparency and size of a virtual arm in VR affected pain perception in 19 patients with CRPS or peripheral nerve injury. Transparency adjustments reduced pain in patients with CRPS but worsened it in those with peripheral nerve injury, while size changes slightly aggravated pain in those with CRPS. These results emphasize the importance of customizing VR interventions to the underlying pathology, further illustrating the interplay between BR and pain experience.

#### Virtual BR for PLP and Amputation-Related Pain

Recent evidence highlights the combined benefits of VR neurorehabilitation in reducing PLP through the integration of visual and tactile feedback. The studies by Ichinose et al [40] and Osumi et al [41] underscored the importance of personalized treatments tailored to the unique characteristics of pain, resulting in significant (effect size=0.68 and 0.87) improvements in both pain intensity and mobility. These findings suggest that using sensory feedback mechanisms within VR environments could enhance rehabilitation outcomes, paving the way for more customized therapeutic approaches.

Ichinose et al [40] investigated the effects of tactile feedback on the cheek during VR-based neurorehabilitation for PLP. Their study involved 9 patients with phantom pain in the upper limbs, who performed exercises mimicking the movements of a virtual limb. In the “cheek condition,” where tactile feedback was applied to the cheek during virtual object interaction, pain intensity significantly (effect size=0.68) decreased compared to the “intact hand condition” and “no stimulus condition.” Even patients without referred sensations experienced notable pain reduction. These findings demonstrate that combining somatosensory and visual feedback can amplify the pain-relieving effects of VR rehabilitation, making it a promising treatment for PLP.

Osumi et al [41] further explored the relationship between PLP characteristics and the efficacy of VR rehabilitation. Their study included 19 patients with PLP who participated in a 20-minute VR session where a virtual arm mimicked phantom limb movement in a mirror-reversed manner. The intervention significantly reduced PLP intensity and improved movement representation (effect size=0.87 and 0.83). A factor analysis revealed 2 distinct types of pain: somatosensory-related pain (eg, burning) and kinesthesia-related pain (eg, clamping). Notably, relief from pain was strongly associated with kinesthetic traits, while no significant correlation was observed with somatosensory characteristics. These results highlight the importance of addressing the kinesthetic aspects of PLP in VR-based rehabilitation programs to optimize therapeutic outcomes.

## Discussion

### Principal Findings

This review represents the first comprehensive analysis of the use of VR as a tool for body remapping in patients with neurological conditions, offering new insights into its potential to manage CNP. Existing studies demonstrate how VR leverages multisensory integration, including visual, tactile, and proprioceptive inputs, to enhance BR and reduce pain [43-45]; for instance, VR-induced leg illusions in individuals with SCI [46] and the manipulation of visual representations of the affected limbs in patients with CRPS reveal how sensory reconfiguration can alleviate pain and reshape BR [47,48]. These findings highlight VR’s capacity to disrupt maladaptive neural pathways and provide a drug-free alternative for pain management [49,50]. Combining VR with SCS enhances pain relief in SCI, while in CRPS, VR with biometric indicators such as heart rate variability reduces pain and modulates responses [45,51-54]. In PLP, kinesthetic inputs outperform sensory cues for rehabilitation [51,55]. Advances in PPS rehabilitation emphasize motor input and sensory awareness for recovery [56]. These findings highlight the role of VR as a transformative tool for neurorehabilitation [46].

Table 5 provides a comprehensive overview of the clinical applications of VR, detailing its benefits across neurological conditions and highlighting its capacity to integrate technology with therapeutic outcomes.

As stated previously, chronic pain involves maladaptive neuroplasticity, altered sensory pathways, and disrupted sensorimotor integration [57]. VR demonstrates significant potential in addressing these mechanisms by recalibrating distorted BR and promoting neuroplasticity [58]. VR reshapes the brain’s BR, reducing pain and improving functionality through integrating visual, tactile, and proprioceptive feedback within immersive environments [59,60]. This approach has been particularly effective in conditions such as CRPS, where VR reduces pain and associated disabilities by correcting altered BR [52,61].

For PLP, VR simulations of the missing limb have shown remarkable success in facilitating cortical reorganization and fostering sensorimotor synchrony, significantly alleviating pain [49]. VR also enhances PPS, aiding sensory and motor recovery in individuals with SCI and amputations [45,62]. VR enables highly personalized treatments that sustain neuroplasticity and enhance long-term therapeutic outcomes, incorporating biofeedback systems such as heart rate variability and spinal cord activity [54,63].

Beyond its physical benefits, VR addresses the emotional and cognitive dimensions of pain by engaging brain regions such as the insular cortex and anterior cingulate cortex [64]. For these reasons, immersive environments could promote the reduction of pain perception, improve emotional regulation, and foster attentional control, actively involving patients in their recovery process [65]. Furthermore, by creating personalized, adaptive interventions informed by real-time physiological data, VR refines its therapeutic effects, offering a robust platform for managing complex pain conditions [54]. These features underscore VR’s capacity to integrate multidimensional pain management strategies, paving the way for its expanded use in clinical practice.

**Table 5:** Descriptions and advantages of virtual reality (VR) therapies on neurological conditions.

Types of VR therapies	Mechanism of action	Intervention duration and number of sessions (based on findings)	Neurological conditions (based on findings)	Benefits and practical application of the technique	Body remapping after virtual therapy
VR and SCS^a^ combination (Solcà et al [12])	Synchronization of SCS-induced paresthesia with VR feedback	Repeated sessions; strong analgesic carryover	Chronic pain (eg, after SCI^b^)	Significant pain reduction; long-lasting effects; reliable “digiceutical” option	Strengthened link between motor and visual sensory feedback mechanisms
Immersive VR for CRPS^c^ (Lewis et al [8]; Solcà et al [42])	Alters visual appearance of affected limb to reduce body perception disturbance	Single to multiple sessions	CRPS and treatment-resistant CRPS	Enhances limb perception, practical for nonpharmacological chronic pain treatment	Improved limb representation; greater spatial awareness
VBS^d^ (Hwang et al [7])	Mental rehearsal with altered body perception; focuses on immersive, realistic experiences	Single session; further studies suggest stronger effects with repetition	CRPS	Enhances body awareness, supports visual-motor integration for immersive VR	Better integration of spatial and kinesthetic awareness
Virtual arm transparency modulation (Matamala-Gomez et al [9])	Adjusting limb appearance in VR to alter pain and perception	Not specified	CRPS and peripheral nerve injury	Tailored interventions based on condition-specific pain mechanisms	Modifies body schema linked to transparency or proportionality cues
VR for PLP^e^ (Ichinose et al [40]; Osumi et al [41])	Mimics virtual limb movement and incorporates tactile feedback to reduce phantom sensations	20-min sessions	PLP	Reduces PLP intensity and restores movement representation; unique integration of visuotactile inputs	Enhanced internal body schema; improved kinesthetic and sensory feedback

^a^SCS: spinal cord stimulation.

^b^SCI: spinal cord injury.

^c^CRPS: complex regional pain syndrome.

^d^VBS: virtual body swapping.

^e^PLP: phantom limb pain.

### Clinical Challenges and Practical Barriers of VR

Despite the promising potential of VR, several practical challenges must be addressed to ensure its effective integration into clinical practice. A significant obstacle is the individual variability in patient response to VR therapy. Factors such as age, cognitive capacity, technological familiarity, and psychological characteristics can influence patient engagement and therapeutic outcomes, potentially affecting the overall effectiveness of the intervention.

Another challenge is cybersickness, which can cause symptoms such as nausea, headache, dizziness, and disorientation, particularly in sensitive and predisposed individuals or during prolonged VR sessions. This side effect can reduce patient adherence and must be mitigated through appropriate system calibration and careful control of session duration.

Moreover, accessibility remains a major concern, especially in resource-limited settings. The high costs associated with hardware, software development, and technical support can prevent the widespread adoption of VR-based interventions. To address these barriers, future developments should focus on creating cost-effective VR solutions, including affordable headsets, mobile-based apps, and open-source platforms that reduce financial barriers for health care providers. Investments in infrastructure and simplified device usability through intuitive interfaces are also essential to ensure equitable access [66].

The requirement for specialized clinical expertise further complicates VR integration. Medical staff must undergo specific training to ensure the proper use of VR systems, maximize therapeutic benefits, and safeguard patient safety. Developing comprehensive training programs for clinicians will be vital to facilitate the widespread adoption of VR-based interventions while minimizing reliance on specialized technical support. Addressing these challenges will require ongoing collaboration among clinicians, researchers, and technology developers to refine VR systems; minimize adverse effects; and ensure that VR remains a practical, accessible, and effective tool for diverse patient populations.

### Future Directions and Clinical Applications

The future of VR in pain management lies in expanding its applications to underexplored conditions and integrating cutting-edge technologies such as real-time neurophysiological monitoring, artificial intelligence (AI), and machine learning. AI is increasingly being applied in the field of rehabilitation, from assessment to treatment. Regarding rehabilitation treatment, AI-based applications can be implemented in the context of the metaverse—a virtual space in which users can interact with a computer-generated environment and other users [67]. In this context, AI applications can be used for motor assessment and for generating virtual character movements to enhance user engagement and enjoyment. These advances could enable the development of more precise and personalized interventions while providing deeper insights into pain mechanisms through adaptive and patient-specific feedback systems.

Moreover, due to its characteristics, VR can easily be combined with other targeted treatments, such as manual therapy, to alleviate chronic pain conditions.

Future studies should investigate the integration of VR with manual therapy because it may enhance chronic pain management through the modulation of pain perception, improving patient engagement and facilitating movement retraining in a controlled environment. However, successful implementation requires therapist expertise, patient suitability, and a strong therapeutic relationship to maximize benefits safely [68]. Furthermore, future research should prioritize large-scale longitudinal clinical trials to assess the long-term efficacy of VR interventions and establish standardized protocols that can be applied across different patient populations. In addition, identifying specific subgroups of patients more likely to benefit from VR therapy could enable more personalized and targeted treatment approaches.

By leveraging advances in immersive technologies and fostering interdisciplinary collaboration, VR could improve therapeutic and diagnostic strategies for different chronic pain conditions, including CNP. If financial, logistical, and clinical barriers are adequately addressed, VR has the potential to become a widely accessible, affordable, and evidence-based tool for pain management across various health care settings.

### Strengths and Limitations of the Review

This review presents a comprehensive and qualitative synthesis of current evidence on the use of VR in pain management. It highlights the potential of VR-based interventions to recalibrate BR, alleviate pain, and foster neuroplastic changes. The strengths of this review lie in its analysis of diverse applications of VR across conditions such as CRPS, PLP, and SCI, providing insights into VR’s therapeutic possibilities. In addition, the inclusion of biofeedback and adaptive technologies reflects the growing emphasis on personalized treatment approaches.

However, several limitations must be acknowledged. This review is based solely on a qualitative analysis, which, while providing valuable descriptive insights, does not allow for a statistical evaluation of effect sizes or direct comparisons between studies. As a result, our review provides a comprehensive qualitative synthesis of the available evidence, offering valuable insights into the field of VR applications for specific chronic pain conditions, identifying key implications for clinical practice and considerations for future investigation.

Moreover, the GRADE assessment revealed variations in the quality of evidence across the included studies. The strongest evidence was found for the outcome of pain reduction, with most of the studies (7/10, 70%) providing moderate-quality evidence, despite some concerns regarding risk of bias and imprecision. The evidence supporting improvements in BR and functional outcomes was generally of moderate to low quality, primarily due to the heterogeneity of intervention protocols and patient populations. The weakest evidence was found for adherence to VR therapy because most of the studies (6/10, 60%) were observational and had a high risk of bias and inconsistency. These findings highlight the need for future high-quality, standardized RCTs with larger sample sizes to confirm the long-term benefits of VR interventions for CNP management. Indeed, many of the included studies were conducted with small sample sizes and heterogeneous methodologies, limiting the generalizability of the findings. Furthermore, the lack of standardized VR protocols complicates the comparison of results across studies. Future research should prioritize longitudinal clinical trials to assess whether the benefits of VR are sustained over extended periods. Given the persistent nature of chronic pain, it is essential to determine whether VR-induced relief remains effective over months or years, thus providing stronger evidence for its integration into long-term pain management strategies. Indeed, the lack of large-scale, standardized clinical trials limits definitive conclusions about the widespread applicability of VR-based interventions. Moreover, potential placebo effects and uncontrolled variables are a challenge in evaluating the true efficacy of VR-based interventions. Accessibility remains an issue because high technological demands and training requirements could hinder widespread implementation, particularly in low-resource settings.

Finally, another limitation of this review is the exclusion of studies focusing on migraines. While this decision was made to maintain a clear methodological focus on conditions where BR mechanisms are central, it might limit the applicability of the findings to other neurological conditions with different pathophysiological mechanisms. Future research could explore the role of VR in migraine treatment to expand the understanding of its broader applicability in neurological care.

### Conclusions

In conclusion, this review highlights VR’s transformative potential in managing CNP. VR could offer a noninvasive, personalized therapeutic approach that recalibrates BR and promotes neuroplasticity by integrating sensory, emotional, and cognitive dimensions. Although challenges such as accessibility and methodological limitations remain, VR could represent a revolutionary tool in pain management in the neurological field.

## Appendix

Multimedia Appendix 1 PRISMA (Preferred Reporting Items for Systematic Reviews and Meta-Analyses) checklist.

## Abbreviations

AI: artificial intelligence
BPD: body perception disturbance
BR: body representation
CNP: chronic neuropathic pain
CRPS: complex regional pain syndrome
GRADE: Grading of Recommendations, Assessment, Development, and Evaluation
MeSH: Medical Subject Headings
PICO: Population, Intervention, Comparison, and Outcomes
PLP: phantom limb pain
PPS: peripersonal space
RCT: randomized controlled trial
RoB 2: revised Cochrane Risk of Bias Tool for Randomized Trials
ROBINS-I: Risk of Bias in Nonrandomized Studies of Interventions
SCI: spinal cord injury
SCS: spinal cord stimulation
VR: virtual reality
